# Effects of 4-Week Complex Decongestive Therapy in the Management of Breast Cancer-Related Arm Lymphedema in Montenegrin Women Post-Mastectomy and Chemo/Radiotherapy

**DOI:** 10.3390/healthcare13202596

**Published:** 2025-10-15

**Authors:** Miloš Kuzmanović, Dušan Mustur

**Affiliations:** 1 Polyclinic “Nova Medic”—Podgorica, Mitra Bakića 148, 81000 Podgorica, Montenegro; kmilos1995@gmail.com; 2 Faculty of Medicine, University of Montenegro, 85347 Igalo, Montenegro

**Keywords:** breast cancer, arm lymphedema, complete decongestive therapy, functional ability, motor strength

## Abstract

**Objectives:** In this study, we aimed to assess the effects of complete decongestive therapy (CDT) on reducing lymphedema and enhancing gross motor strength (GMS), functional ability in the upper arm, quality of life (QoL), and pain relief among women who had undergone breast cancer surgery and chemo/radiotherapy in Montenegro. **Methods**: This prospective observational/pilot study included 50 women with breast cancer-related arm lymphedema, with an average age of 60.88 ± 12.78 years. The four-week Phase1-CDT program involved manual lymphatic drainage, compression bandaging, skin care, tailored kinesitherapy and patient education. Measurements included arm edema circumference compared to the contralateral arm, pain severity (VAS), arm muscle strength (MMT), functional ability (QDASH), and overall QoL (WHOQOL-BREF). **Results**: Following CDT, significant reductions in lymphedema circumference were observed in various areas and overall (*p* = 0.002), along with improvements in overall upper-arm GMS (*p* = 0.002) and specific upper-extremity movements such as wrist and forearm flexion, supination, and external rotation (*p* < 0.001). Significant improvements were also observed in pain severity and QDASH scores (*p* < 0.001), and overall QoL significantly increased (*p* < 0.001). Muscle strength in the hand, wrist, forearm, and shoulder also improved significantly (*p* < 0.05). We found a negative correlation between edema size and motor function in different muscle groups of the upper extremities, as well as between the QDASH score, quality of life, and overall upper-arm gross motor strength. **Conclusions**: It was observed that the four-week Phase 1-CDT program significantly improved lymphedema severity, functional abilities, gross motor strength, quality of life, and pain levels in Montenegrin women with breast cancer who had undergone mastectomy and chemo/radiotherapy. Our findings are limited to the immediate post-intervention period. This study is the first of its kind in Montenegro, suggesting the need for future randomized studies with a larger number of participants are needed.

## 1. Introduction

Breast cancer is a significant health concern for women in Montenegro, as it is the most common cancer and a leading cause of cancer-related deaths. Despite the benefits of early detection through screening programs, breast cancer remains a major issue in Montenegro, with a higher mortality rate compared to the European average [1]. The choice of treatments and cancer survival rates depend on the stage of the cancer at the time of diagnosis [2]. Currently, Montenegro does not have a national registry for breast cancer patients. As a result, statistics from the Global Cancer Observatory (GLOBOCAN) are primarily relied upon [3,4]. In Montenegro, a small country with only 600,000 inhabitants, approximately 400 new cases of breast cancer are diagnosed each year, resulting in over 110 deaths [3].

Breast cancer-associated arm lymphedema (BCRL) poses a lifelong risk for survivors and can become a lifelong burden once acquired. While there are no specific data available for Montenegro, approximately 20% of women develop secondary lymphedema after cancer treatment [5]. However, the incidence of lymphedema can vary widely, from 2% to 77%, depending on the specific local-regional and systemic treatments used [6]. Factors such as undergoing a modified radical mastectomy with removal of most of the lymph nodes in the underarm area (axillary lymph nodes), receiving chemotherapy and/or radiotherapy and having a higher body mass index (BMI), are linked to a higher risk of developing BCRL [7,8,9,10,11].

The majority of cases of secondary lymphedema in breast cancer survivors occur in the first few years following surgery, with around ¾ occurring within the first year and 90% within 3 years [12]. Lymphedema occurs due to the lymphatic system’s impaired function, resulting in an abnormal fluid buildup in the arm causing swelling, limited physical ability and discomfort [8,9,10,11]. Early detection and proper management are essential to prevent arm swelling progression and enhance the quality of life for breast cancer survivors [13,14].

Arm lymphedema remains a challenging condition for both breast cancer survivors and healthcare practitioners, significantly affecting patient functioning, the muscle strength of the affected arm and quality of life. There is currently no proven pharmacological treatment for treating secondary arm lymphedema in breast cancer survivors. Complete decongestive therapy (CDT) is a key intervention in physical rehabilitation that provides numerous benefits. These benefits include reducing swelling, enhancing arm function, increasing muscle strength, alleviating pain, and improving overall quality of life (QoL) [15]. The Phase1-CDT program, known as the “intensive phase,” consists of continuous multilayer bandage compression therapy, exercises, and multiple weekly manual lymphatic drainage sessions. Treatment sessions typically last between 2 and 4 weeks with the goal of moving lymphatic fluid to reduce arm swelling. The Phase2-CDT program, known as the “maintenance phase,” continues at home after the intensive phase, where patients continue techniques learned to manage their condition and prevent edema recurrence [13,15]. Alternative treatments like reflexology, acupuncture, acupressure, and photo-biomodulation therapy have not yielded conclusive outcomes in the management of BCRL [16].

The World Health Organization (WHO) predicts a 47% increase in new cancer cases by 2040 [3]. In Montenegro, the main focus of cancer management is on screening programs, but there is a lack of attention to the needs of breast cancer survivors who develop secondary lymphedema. This is concerning, especially considering that disability rates are expected to rise in Montenegro and the Western Balkan region by 2030 [17].

Klassen O et al. (2017) noted a connection between lower gross motor strength in the upper limb and a higher mortality risk among breast cancer survivors [18]. Advancements in early diagnosis, patient education, expert consensus, and novel treatments are essential for preventing and managing BCRL. Lymphatic rehabilitation following mastectomy and chemo/radiotherapy plays a vital role in this process. Early-stage arm lymphedema shows better response to treatment compared to advanced stages with fibrosis, which may require more aggressive interventions [19,20].

Age and obesity are factors that can impact lymphedema outcomes, especially in middle-aged and older women with breast cancer [8,11,19].

Lymphatic rehabilitation services in Montenegro are currently limited, making it difficult to effectively manage lymphedema, especially in breast cancer patients. The lack of a registry hinders planning for oncological interventions and rehabilitation. The country must strengthen its healthcare system to offer cost-effective early rehabilitation for all patients with arm lymphedema, improving their quality of life and potentially extending survival. Patients who are denied specialized medical rehabilitation can appeal to the Ministry of Health, leading to further delays in starting the CDT program.

Our prospective observational/pilot study aims to assess the effects of complex decongestive therapy on arm lymphedema in Montenegrin women who have undergone mastectomy and chemo/radiotherapy for breast cancer. In Phase 1-CDT, we will explore the correlation between edema size and motor strength. We will also assess the enhancements in functional disability, quality of life, pain levels, and motor strength. Despite the limited sample size, this study aims to provide valuable insights for healthcare practitioners treating lymphedema in breast cancer survivors.

## 2. Materials and Methods

A single-center, longitudinal study was carried out at the Regional Health Center in Danilovgrad from April 2023 to June 2024. The study involved 50 women with a median age of 53.3 years who had undergone surgery and received adjuvant breast cancer chemotherapy or radiotherapy within the past 6–36 months.

Patients eligible for the study had to meet specific criteria, stating that (1) they had undergone modified radical mastectomy with lymph node removal and had received radiotherapy and/or chemotherapy; (2) they required physiotherapy treatment as recommended by a physician; and (3) they had not previously undergone any physical therapy procedures following mastectomy. The participants’ demographic information, including sex, age, education levels, marital status, comorbidities and medication use, was collected. Exclusion criteria for the study comprised the presence of neurological or mental illnesses that could affect exercise, uncontrolled cardiopulmonary diseases (e.g., heart failure and chronic obstructive pulmonary disease), deep vein thrombosis or the use of anticoagulant therapy, and withdrawal from the study for any reason. Participants who did not provide all the necessary data on two occasions were excluded as well. In this prospective observational/pilot study, we did not perform formal power calculations to determine the sample size. The primary goals were to evaluate feasibility, refine methods, and gather initial data specific to the Montenegrin population. The sample size was determined based on practical considerations such as recruitment difficulties in a small country and budget limitations. Participants were enrolled consecutively to reduce selection bias. The study was conducted in accordance with the Declaration of Helsinki. The Ethics Committee of the Regional Health Center “Dimitrije Dika Marenić” in Danilovgrad approved the study (Protocol No. 829, issued on 21 March 2023). All participants provided written consent.

All analyses were performed using the Statistical Package for the Social Sciences for Windows (IBM SPSS Statistics, Chicago, IL, USA), version 26.0 software program. A significance level of *p* < 0.05 was employed in this study. The normality of the distribution was assessed using the Wilk-Shapiro test. In cases where deviations from normal distribution were identified, non-parametric statistical tests were applied to the variables. The χ^2^ test was employed to determine statistically significant discrepancies in categorical variables among the sample. The Wilcoxon test was used for continuous variables. When comparing groups, such as before and after the physiotherapy procedure, Pearson’s χ2 test was used for categorical variables, and Wilcoxon’s paired rank test was used for continuous variables. Spearman’s correlation coefficient Rho (R) was utilized to evaluate correlations due to the deviations from normal distribution.

### 2.1. Lymphatic Rehabilitation Program

The *CDT program* is typically divided into two phases. Phase 1 of a CDT program for breast-cancer-related lymphedema aims to actively reduce swelling and improve symptoms in patients with moderate to severe lymphedema. It includes manual lymphatic drainage, compression bandaging, proper skin care, tailored kinesitherapy and patient education. Foam bandages are worn almost continuously and only removed for personal hygiene. Treatment sessions typically last between 2 and 4 weeks, 5 days a week, with each session lasting about an hour. These core components are tailored to individual patient needs and tolerance levels [19,21].

*Manual lymphatic drainage (MLD)* is a crucial part of the program to reduce muscle spasm and swelling and improve lymphatic flow. It is a gentle technique that helps move lymphatic fluid away from the affected area, aiming to improve the function of the lymphatic system by redirecting fluid around blockages in the body. MLD involves gentle skin stretching, slow rhythmic massage, and deep breathing to promote a healthy lymphatic system and encourage fluid movement from blocked areas back into the body [22].

*Compression therapy* involves using multilayered foam bandages on the affected upper limb to reduce fluid buildup. These bandages, along with foam, apply gentle pressure to prevent reaccumulation, and are worn continuously, only being removed for hygiene purposes [19].

*Proper skin care* of the affected area is essential to prevent infections, including maintaining good hygiene and moisturizing the skin with lotion to keep it clean and healthy [22].

*The customized kinesitherapy* program was conducted 5 days a week. The program focused on specific exercises aimed at boosting lymphatic drainage and enhancing mobility, with the primary goal of facilitating the movement of fluid out of the affected body area. The exercise regimen was tailored to the individual’s needs, considering their symptoms, age, and underlying health issues. Tailored kinesitherapy for breast cancer survivors is highly beneficial, improving physical and psychological well-being, reducing the risk of disease recurrence, and extending survival [19,23,24,25,26,27,28].

*Patient education* focuses on teaching patients how to control factors such as diet, stress, weight, and the importance of conducting Phase 2-CDT for long-term management. Phase 2-CDT involves self-care practices like self-massage, wearing compression garments, exercising, and proper skin care to prevent infections like cellulitis [19,29,30,31].

### 2.2. Outcomes

The assessment of *secondary lymphedema of the upper limb* involved comparing limb circumferences at different levels and considering the duration of lymphedema. The stages of lymphedema were classified by the International Society of Lymphology from 0 to 3, based on the softness or firmness of the limb and the response to elevation. Severity within stages 1 to 3 was determined by the percentage increase in volume: mild (<20%), moderate (20–40%), or severe (>40%). Stage 0 represented a subclinical condition with impaired lymphodynamics but no visible swelling [23,32].

*Gross motor strength* (GMS) was assessed by conducting manual muscle testing (MMT) on twelve muscle groups/motions. The overall upper-arm GMS was also evaluated, with grades ranging from 5 (normal strength) to 0 (no contraction palpable). Overall upper-arm GMS refers to the general capacity of the muscles in the upper arm (biceps, triceps, brachialis, and coracobrachialis) to exert force and power during functional movements. Assessment begins at grade 3, indicating the ability to move through the full range of motion against gravity and may progress or decrease from there [33].

*Body mass index (BMI)* was classified as: normal (18.5–24.9), overweight (25–29.9) or obese (30 and over) [10].

*The Quick Disabilities of the Arm, Shoulder, and Hand (QDASH)* questionnaire was used to assess upper extremity disability, with scores ranging from 0 (no disability) to 100 (most severe disability) [34].

The researchers utilized the Serbian version of the *World Health Organization’s Quality of Life (WHOQOL-BREF)* scale to evaluate the overall quality of life in patients, with scores converted to a 0–100 scale, indicating different levels of quality of life [35,36,37].

*Pain severity* was measured using the visual analog scale (VAS, 0–10) on two occasions to track changes in pain intensity after CDT.

## 3. Results

This study included 50 female participants who had undergone radical non-sparing mastectomy for breast cancer. The average age of the participants was 60.88 ± 12.78 years, with most having incomplete education from high school (60.00%). The majority of the participants were in the 60–69 age group, living with their families (48.00%) or husbands (34.00%), with over half residing in urban areas. Most patients were diagnosed with stage 2 carcinoma. All the participants had undergone radical non-sparing mastectomies and were afflicted with unilateral arm lymphedema, with the left upper arm being the most affected site. Surgery was followed by chemotherapy in 88% and radiotherapy in 70% of cases. More than half of the participants had stage 2 lymphedema. The average time from lymphedema development was 26.18 ± 7.78 months. Many participants had significant comorbidities, such as osteoarthritis (82.00%) and systemic arterial hypertension (78.00%). Additionally, 28.00% had diabetes mellitus and 14.00% had hypothyroidism. The time from surgery to starting the CDT program was typically one to two years for 64.00% of participants. Regarding body weight, 20.00% were normal weight, 56.00% were classified as overweight, and 24.00% were obese based on BMI classification (see Table 1 for detailed results).

Complete decongestive therapy is the main treatment for arm lymphedema, which can cause arm swelling and pain in breast cancer survivors. This condition can impact muscle strength, physical function, and overall quality of life. Addressing these issues is crucial for improving the overall health of these patients. Significant reductions in lymphedema were observed at the level of the metacarpophalangeal joints (SMD 2.01, 95% CI 1.12–2.90), radial styloid process (SMD 2.09, 95% CI 1.18–3.00), 10 cm proximal to the radial styloid process (SMD 0.61, 95% CI −0.04–1.26), 20 cm proximal (SMD 0.69, 95% CI 0.03–1.35), 30 cm proximal (SMD 0.75, 95% CI 0.09–1.42), and olecranon level (SMD 1.31, 95% CI 0.56–2.05). Improvements were observed at all levels except 40 cm proximal to the radial styloid process (SMD 0.26, 95% CI −0.37–0.88). At this specific level, the *p*-value was exactly 0.05, leading to the failure to reject the null hypothesis, suggesting that the result is not statistically significant. This does not mean the null hypothesis is true; it simply means there is not enough evidence to reject it (Table 2).

Patient-reported pain intensity significantly decreased after the four-week complete decongestive therapy program (SMD 1.70, 95% CI 0.88–2.52) (Table 3).

Overall gross motor strength of the upper arm increased significantly from 3.30 ± 0.70 at the start to 4.10 ± 0.50 at discharge, with improvements observed in all upper-arm movements. Significant improvements in gross motor strength were observed in hand flexors (SMD −1.00, 95% CI −1.70 to −0.30), hand extensors (SMD −1.06, 95% CI −1.77 to −0.36), wrist flexors (SMD −2.41, 95% CI −3.40 to −1.43), wrist extensors (SMD −1.38, 95% CI −2.14 to −0.62), forearm flexors (SMD −2.41, 95% CI −3.40 to −1.43), forearm extensors (SMD −0.93, 95% CI −1.62 to −0.24), supinators (SMD −2.60, 95% CI −3.63 to −1.57), pronators (SMD −1.38, 95% CI −2.14 to −0.62), shoulder adductors (SMD −0.74, 95% CI −1.40 to −0.07), shoulder abductors (SMD −1.23, 95% CI −1.96 to −0.49), internal rotators of the upper arm (SMD −0.87, 95% CI −1.55 to −0.19), external rotators of the upper arm (SMD −0.99, 95% CI −1.68 to −0.29), and overall upper-arm gross motor strength (SMD −1.32, 95% CI −2.06 to −0.57) (Table 4).

Significant improvements were observed in functional upper extremity disability (QDASH, SMD 1.33, 95% CI 0.58–2.08) and overall quality of life (WHOQOL-BREF, SMD −3.74, 95% CI −5.09 to −2.40) after the four-week complete decongestive therapy program. The QDASH score decreased from 64.40 ± 21.60 at admission to 40.50 ± 13.50 at discharge. The WHOQOL-BREF score improved from 43.80 ± 10.41 at admission to 84.02 ± 11.06 at discharge (Table 5).

The study also explored the relationship between upper extremity edema size and gross motor strength in different muscle groups of the upper extremity post-treatment, revealing a negative correlation between edema size and motor function in all observed muscle groups (Table 6).

Furthermore, positive associations were found between upper extremity functional disability score, pain level, and the overall arm lymphedema size. A negative correlation was observed between the upper extremity functional disability score, quality of life questionnaire score, and overall arm gross motor strength (Table 7).

## 4. Discussion

In our study, we observed that a four-week Phase 1-CDT program significantly improved lymphedema severity, functional abilities, gross motor strength, quality of life, and pain levels in Montenegrin women with breast cancer who had undergone mastectomy and chemo/radiotherapy.

This study is the first of its kind in Montenegro and highlights the benefits of such a program for breast cancer survivors. Healthcare challenges in Montenegro, including limited access to screening and diagnostics, shortages of trained personnel, inadequate health education, and weak infrastructure, have a significant impact on breast cancer survival rates. Additionally, the late presentation of women for screening further complicates the situation. The absence of a cancer registry also hampers the planning of oncological interventions and rehabilitation efforts. To address these issues, Montenegro needs to enhance its healthcare system to provide early rehabilitation for all patients with arm lymphedema, which could improve their quality of life and potentially extend their survival.

While this study is a prospective observational/pilot study with a small sample size, which may limit generalizability, our findings align with randomized controlled trials that support CDT as an important procedure for breast cancer survivors [6,15,19,21,38]. Previous studies often did not fully address the functional issues faced by breast cancer survivors with arm lymphedema.

In our study, we found a significant decrease in the arm volume difference between the affected and healthy arm after participants completed the CDT program. The improvements were noticeable at all levels except 40 cm proximal to the radial styloid process. At this specific level, the *p*-value was 0.05, indicating that the result was not statistically significant. This does not confirm the null hypothesis but suggests that there is not enough evidence to reject it. Samanci N. et al. (2019) demonstrated significant improvement in lymphedema volume after CDT treatment [39]. Borman P et al. (2022) also reported positive outcomes, including improved functional status, quality of life and reduced lymphedema volume [40]. Our study went further to evaluate pain relief and muscular strength improvement in different arm muscle groups, providing additional valuable insights. Sezgin Ozcan D. et al. assessed the effects of four weeks of CDT on various aspects but did not include an evaluation of gross motor strength in different muscle groups of the upper limb [41].

This study is the first to investigate the effects of complete decongestive therapy on arm lymphedema among Montenegrin women post-mastectomy and chemo/radiotherapy.

Factors such as radical mastectomy, chemotherapy, radiotherapy, age, obesity, hypertension, osteoarthritis, and diabetes mellitus can influence the effects of CDT in populations like those in our study [12]. Severe lymphedema was more common in patients who underwent radical mastectomy [42].

In our study, the average age of the participants was 60.88 ± 12.78 years, with a majority being overweight (56%) or obese (24%) based on their BMI. Concomitant conditions such as hypertension, osteoarthritis and diabetes mellitus can have a negative impact on lymphedema size and treatment outcomes. These conditions can worsen the underlying conditions treated by CDT and affect a patient’s ability to tolerate and benefit from the therapy. Healthcare providers should address and manage these comorbidities alongside CDT to optimize patient outcomes. The intake of medications and the influence of chronic diseases on the lymphatic system can contribute to unfavorable results [43]. However, in our research, these factors did not significantly affect the outcomes.

A recent review by Gilchrist L et al. (2024) analyzed 13 systematic reviews on the effects of CDT for arm BCRL. The review highlighted inconsistencies as a major issue in the implementation and results of the therapy, emphasizing the need for standardized staging criteria and outcome measures. Future studies should prioritize consistent, clinically relevant, and achievable outcomes, particularly in relation to reducing arm lymphedema [44].

Standardized outcome measures are essential for evaluating and managing secondary lymphedema among breast cancer patients. A core outcome set has been developed recently, including important domains such as lymphedema stage, volume, pain, and patient-reported outcomes related to quality of life and function, similar to our study [45,46]. In our research, assessment tools for breast cancer related lymphedema include circumferential measurements using a tape measure, manual muscle testing, pain level measurement using a visual analog scale, assessment of muscle strength in the affected arm through manual muscle testing, patient-reported upper quadrant function using QuickDASH, and patient-reported quality of life [46].

Similarly to Borman P et al.’s study, the majority of our patients (78%) had stage 1 or stage 2 lymphedema, characterized by mild to moderate swelling, which could be managed well with elevation and CDT [40]. Seeking treatment at an early stage is important to prevent progression to more severe stages of lymphedema [41,47].

Our study also aimed to investigate the relationships between arm lymphedema, arm gross motor strength, functional disability score, pain, and overall quality of life among breast cancer survivors. We found a significant association between the severity of arm lymphedema and a decline in quality of life, consistent with previous research [48]. Additionally, we observed a negative correlation between edema size and motor function in different muscle groups of the upper extremity, indicating a potential link between impaired lymph drainage and decreased motor skills. Our study also revealed positive correlations between QDASH score and pain levels, as well as negative correlations between the QDASH score, quality of life and overall upper-arm gross motor strength. These findings align with the existing literature, but further research is needed to fully understand the complex relationships among these variables [49].

## 5. Limitations

This study has limitations, including the absence of a control group and a small, non-randomized sample, which may affect the generalizability of the findings. Recruiting 50 participants among breast cancer survivors with secondary lymphedema in a small country like Montenegro was challenging. Despite these limitations, this study is the first to investigate the effects of complete decongestive therapy on arm lymphedema in Montenegrin women post-mastectomy and chemo/radiotherapy.

The study aimed to provide all participants with the same Phase 1-CDT program to reduce edema size and pain levels, improve motor strength, functional ability, and quality of life. However, the study did not have a longer follow-up post-rehabilitation program, including adherence to Phase 2-CDT, which is another limitation. Follow-ups at one and three months were not possible due to low response rates. Nevertheless, a retrospective review indicated sustained benefits of CDT for 24 months, particularly in individuals with severe lymphedema [50].

## 6. Conclusions

In our study, the first of its kind in Montenegro, we observed the influence of the four-week Phase 1-CDT program on breast cancer survivors. The results revealed significant improvements in lymphedema, motor strength, and arm function, along with reductions in pain and enhancements in overall quality of life. These positive outcomes were observed across individuals with various factors such as radical mastectomy, chemotherapy, radiotherapy, older age, or significant comorbidities. It is important to note that our findings are limited to the immediate post-intervention period. Additionally, negative correlations were identified between edema size and arm function, as well as between functional disability, arm gross motor strength, and overall quality of life.

These findings underscore the importance of early intervention with comprehensive therapy programs and suggest a need for further research pertaining to Montenegro and the broader Western Balkan region. Regional strategies should prioritize the management of secondary lymphedema and utilize standardized assessment tools to evaluate lymphedema severity, functional disability, arm gross motor strength, pain levels and overall quality of life in breast cancer survivors. This approach can help mitigate the anticipated increase in disability rates in the area.

As mentioned earlier, the absence of a control group or randomization limits the reliability of the findings. Future studies should include a larger sample size and incorporate control groups or randomization to enhance the validity of the results.

## Figures and Tables

**Table 1:** Demographic and other characteristics of the participants.

Variables		*N*	%
***Age groups***	18–39	2	4.00%
	40–49	7	14.00%
	50–59	12	24.00%
	60–69	15	30.00%
	70 and over	14	28.00%
***Education levels***	Without completing primary school	1	2.00%
	Primary school	13	26.00%
	Secondary school	30	60.00%
	Higher education	3	6.00%
	Master’s or PhD degree	3	6.00%
***Marital status***	Single	3	6.00%
	Divorced	11	22.00%
	Married	31	62.00%
	Divorced	0	0.00%
	Widow	5	10.00%
***Concomitant*** ***conditions***	Osteoarthritis	41	82.00%
	Systemic arterial hypertension	39	78.00%
	Diabetes mellitus	14	28.00%
	Hypothyroidism	7	14.00%
	Asthma bronchialis	4	8.00%
***Place of residence***	Rural	22	44.00%
	Urban	28	56.00%
***Stage of breast carcinoma***	Stage 1	9	18.00%
	Stage 2	24	48.00%
	Stage 3	17	34.00%
***Stage of lymphedema***	Stage 1	13	26.00%
	Stage 2	26	52.00%
	Stage 3	11	22.00%
***Body Mass Index***	Normal	10	20.00%
	Overweight	28	56.00%
	Obese	12	24.00%
***Type of treatment received***	Modified radical mastectomy	50	100.00%
	Chemotherapy	44	88.00%
	Radiotherapy	35	70.00%
***Time since surgery***	Up to 1 year	8	16.00%
	1 year to 2 years	32	64.00%
	2 years to 3 years	10	20.00%
***Time since the onset of arm lymphedema***	Up to 1 year	4	8.00%
	1 year to 2 years	12	24.00%
	2 years to 3 years	34	68.00%

**Table 2:** Comparison of edema size in breast cancer survivors before and after a complete decongestive therapy program, assessed by the volume difference between the affected and healthy arms.

Lymphedema Measurements Areas (in Centimeters)	Before Completing the Decongestive Therapy Program	After Completing the Decongestive Therapy Program	Standardized Mean Difference (95% CI)	*p* Value
	x ± SD (Range)			
***L*** ***evel of the metacarpophalangeal joints***	3.12 ± 0.94 (1.00–5.00)	1.27 ± 0.90 (0.10–4.00)	2.01 (1.12, 2.90)	**0.003**
***Level of the Radial styloid process***	3.99 ± 1.51 (0.50–9.00)	1.17 ± 1.17 (0.10–6.00)	2.09 (1.18, 3.00)	**<0.001**
***10 cm proximal to the Radial styloid process***	3.06 ± 2.16 (0.00–8.00)	1.96 ± 1.34 (0.10–5.00)	0.61 (−0.04, 1.26)	0.042 **(*p* < 0.05)**
***2*** ***0 cm proximal to the Radial styloid process***	2.73 ± 1.15 (0.30–5.00)	1.79 ± 1.54 (0.10–7.00)	0.69 (0.03, 1.35)	0.034 **(*p* < 0.05)**
***30 cm proximal to the Radial styloid process***	3.03 ± 1.06 (0.40–5.00)	1.69 ± 2.29 (0.10–10.00)	0.75 (0.09, 1.42)	0.009 **(*p* < 0.01)**
***40 cm proximal to the Radial styloid process***	2.99 ± 1.01 (0.50–5.00)	2.39 ± 3.12 (0.10–4.00)	0.26 (−0.37, 0.88)	0.050
***Olecranon level***	3.12 ± 0.94 (1.00–5.00)	1.21 ± 1.84 (0.10–5.00)	1.31 (0.56, 2.05)	0.022 **(*p* < 0.05)**
***Overall lymphedema size***	3.10 ± 1.06 (0.40–5.00)	1.61 ± 0.79 (0.10–4.00)	2.07 (1.16, 2.95)	**0.002**

**Table 3:** Patient-assessed pain intensity in breast cancer survivors before and after a four-week complete decongestive therapy program.

Variable	Before Completing the Decongestive Therapy Program		After Completing the Decongestive Therapy Program		Standardized Mean Difference (95% CI)	*p* Value
	x ± SD	Range	x ± SD	Range		
***Pain* (VAS)**	**6.14 ± 1.21**	2.00–9.20	3.41 ± 1.92	1.20–7.10	1.70 (0.88, 2.52)	**<0.001**

**Table 4:** Gross motor strength in breast cancer survivors before and after a four-week complete decongestive therapy program.

Gross Motor Strength	Before Completing the Decongestive Therapy Program	After Completing the Decongestive Therapy Program	Standardized Mean Difference (95% CI)	*p* Value
	x ± SD (Range)			
**Hand flexors**	**3.60 ± 0.80 (1.00–5.00)**	**4.40 ± 0.80 (2.00–5.00)**	−1.00 (−1.70, −0.30)	0.021 (***p* < 0.05**)
**Hand extensors**	3.40 ± 0.80 (1.00–5.00)	4.20 ± 0.70 (3.00–5.00)	−1.06 (−1.77, −0.36)	0.014 (***p* < 0.05**)
**Wrist flexors**	3.40 ± 0.70 (2.00–5.00)	4.70 ± 0.30 (2.00–5.00)	−2.41 (−3.40, −1.43)	**<0.001**
**Wrist extensors**	3.30 ± 0.70 (2.00–5.00)	4.20 ± 0.60 (2.00–5.00)	−1.38 (−2.14, −0.62)	0.041 (***p* < 0.05**)
**Forearm flexors**	3.40 ± 0.70 (2.00–5.00)	4.70 ± 0.30 (3.00–5.00)	−2.41 (−3.40, −1.43)	**<0.001**
**Forearm extensors**	3.40 ± 0.80 (2.00–5.00)	4.10 ± 0.70 (2.00–5.00)	−0.93 (−1.62, −0.24)	0.031 (***p* < 0.05**)
**Supinators**	3.30 ± 0.70 (2.00–5.00)	4.70 ± 0.30 (2.00–5.00)	−2.60 (−3.63, −1.57)	**<0.001**
**Pronators**	3.30 ± 0.70 (2.00–5.00)	4.20 ± 0.60 (2.00–5.00)	−1.38 (−2.14, −0.62)	0.041 (***p* < 0.05**)
**Shoulder adductors**	3.20 ± 0.90 (2.00–5.00)	3.90 ± 1.00 (2.00–5.00)	−0.74 (−1.40, −0.07)	0.048 (***p* < 0.05**)
**Shoulder abductors**	3.30 ± 0.70 (2.00–5.00)	4.10 ± 0.60 (2.00–5.00)	−1.23 (−1.96, −0.49)	0.031 (***p* < 0.05**)
**Internal rotators of the upper arm**	3.30 ± 0.70 (2.00–5.00)	4.00 ± 0.90 (3.00–5.00)	−0.87 (−1.55, −0.19)	0.022 (***p* < 0.05**)
**External rotators of the upper arm**	3.30 ± 0.70 (1.00–5.00)	3.90 ± 0.50 (3.00–5.00)	−0.99 (−1.68, −0.29)	**0.001**
**Overall upper-arm gross motor strength**	3.30 ± 0.70 (1.00–5.00)	4.10 ± 0.50 (3.00–5.00)	−1.32 (−2.06, −0.57)	**0.002**

**Table 5:** Functional upper extremity disability score and overall quality of life score in breast cancer survivors before and after a four-week complete decongestive therapy program.

Variable	Before Completing the Decongestive Therapy Program		After Completing the Decongestive Therapy Program		Standardized Mean Difference (95% CI)	*p* Value
	x ± SD	Range	x ± SD	Range		
**QDASH**	**64.40 ± 21.60**	20.00–100.00	40.50 ± 13.50	18.00–80.00	1.33 (0.58, 2.08)	**<0.001**
**WHOQOOL-BREF**	43.80 ± 10.41	15.00–78.00	84.02 ± 11.06	21.00–100.00	−3.74 (−5.09, −2.40)	**<0.001**

**Table 6:** Correlation between the size of upper extremity edema and gross motor strength in various muscle groups in the upper extremity post-treatment.

Gross Motor Strength of Various Muscle Groups in the Upper Extremities		Upper Extremity Edema Size
**Hand flexors**	R	−0.531
	*p*	<0.001
**Hand extensors**	R	−0.600
	*p*	<0.001
**Wrist flexors**	R	−0.568
	*p*	<0.001
**Wrist extensors**	R	−0.442
	*p*	<0.001
**Forearm flexors**	R	−0.567
	*p*	<0.001
**Forearm extensors**	R	−0.455
	*p*	<0.001
**Supinators**	R	−0.484
	*p*	<0.001
**Pronators**	R	−0.442
	*p*	0.004
**Upper-arm adductors**	R	−0.462
	*p*	0.002
**Upper-arm abductors**	R	−0.531
	*p*	<0.001
**Internal rotators of the upper arm**	R	−0.697
	*p*	<0.001
**External rotators of the upper arm**	R	−0.302
	*p*	0.005
**Overall upper-arm gross motor strength**	R	−0.621
	*p*	<0.001

**Table 7:** Correlation between upper extremity functional disability scores, quality of life questionnaire, pain level, and gross motor strength post-treatment.

Variables		Upper Extremity Functional Disability Score (QDASH)
***Quality of life (WHOQOOL)***	*R*	*−0.654*
	*p*	***<0.001***
***Pain (VAS)***	*R*	*0.683*
	*p*	***<0.001***
***Overall lymphedema size***	*R*	*0.697*
	*p*	***<0.001***
***Overall upper-arm gross motor strength (GMS)***	*R*	*−0.670*
	*p*	***<0.001***

## Data Availability

The data from this study can be obtained by contacting the first or corresponding author, as privacy and ethical considerations restrict their public availability.

## Abbreviations

The following abbreviations are used in this manuscript:

CDT	Complete Decongestive Therapy
BCRL	Breast-Cancer-Related Lymphedema
GLOBOCAN	Global Cancer Observatory
BMI	Body Mass Index
WHO	World Health Organization
GMS	Gross Motor Strength
MMT	Manual Muscle Test
QDASH	Quick Disabilities of the Arm, Shoulder, and Hand
MLD	Manual Lymphatic Drainage
QoL	Quality of Life
WHOQOL-BREF	World Health Organization’s Quality of Life-Brief Version
